# Lysophosphatidylcholine Acetyltransferase 2 (*LPCAT2*) Influences the Gene Expression of the Lipopolysaccharide Receptor Complex in Infected RAW264.7 Macrophages, Depending on the *E. coli* Lipopolysaccharide Serotype

**DOI:** 10.3390/biology13050314

**Published:** 2024-05-01

**Authors:** Victory Ibigo Poloamina, Hanaa Alrammah, Wondwossen Abate, Neil D. Avent, Gyorgy Fejer, Simon K. Jackson

**Affiliations:** 1 Faculty of Health, University of Plymouth, Plymouth PL4 8AA, UK; 2 Zoonoses Research Unit, College of Veterinary Medicine, University of Bagdad, Baghdad 10071, Iraq; 3 College of Medicine and Health, University of Exeter, Exeter EX1 2HZ, UK

**Keywords:** CD14, TLR4, MD2, LPCAT2, gene, lipid raft, RAW264.7, macrophage, lipopolysaccharide, *E. coli*

## Abstract

**Simple Summary:**

*E. coli* harms over 100 million patients worldwide annually. Its toxin component is lipopolysaccharide, which has various forms, referred to as smooth or rough, based on their structure. Macrophages’ main function is to eat up foreign organisms through phagocytosis. This process requires membrane fluidity, which means the phospholipid in the macrophage plasma membrane needs to be regularly remodelled. A lipid-modifying enzyme known as LPCAT2 performs phospholipid remodelling and participates in inflammation. Here, we studied how this enzyme influences the gene expression of receptors that recognise and bind to lipopolysaccharides. These receptors are *TLR4, CD14*, and *MD2*. Our results show that LPCAT2 only influenced these receptors when macrophages were infected with smooth lipopolysaccharides. We conclude that LPCAT2 does not influence the gene expression of the receptors in macrophages infected with rough lipopolysaccharides because they do not require lipid-raft microdomains to mediate inflammation, but smooth lipopolysaccharides do.

**Abstract:**

*Escherichia coli* (*E. coli*) is a frequent gram-negative bacterium that causes nosocomial infections, affecting more than 100 million patients annually worldwide. Bacterial lipopolysaccharide (LPS) from *E. coli* binds to toll-like receptor 4 (TLR4) and its co-receptor’s cluster of differentiation protein 14 (CD14) and myeloid differentiation factor 2 (MD2), collectively known as the LPS receptor complex. LPCAT2 participates in lipid-raft assembly by phospholipid remodelling. Previous research has proven that LPCAT2 co-localises in lipid rafts with TLR4 and regulates macrophage inflammatory response. However, no published evidence exists of the influence of LPCAT2 on the gene expression of the LPS receptor complex induced by smooth or rough bacterial serotypes. We used RAW264.7—a commonly used experimental murine macrophage model—to study the effects of LPCAT2 on the LPS receptor complex by transiently silencing the *LPCAT2* gene, infecting the macrophages with either smooth or rough LPS, and quantifying gene expression. LPCAT2 only significantly affected the gene expression of the LPS receptor complex in macrophages infected with smooth LPS. This study provides novel evidence that the influence of LPCAT2 on macrophage inflammatory response to bacterial infection depends on the LPS serotype, and it supports previous evidence that LPCAT2 regulates inflammatory response by modulating protein translocation to lipid rafts.

## 1. Introduction

**Figure 1:**
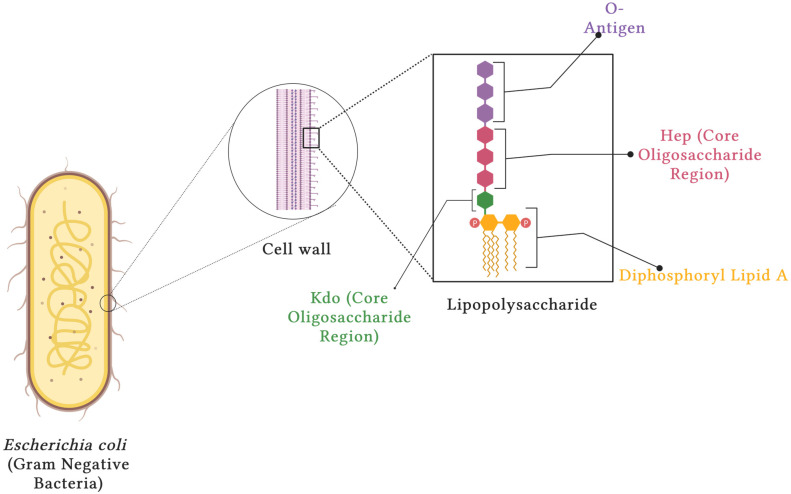
General structure of bacterial lipopolysaccharide [5,6,7,8]. Created with BioRender.com (web-based illustration software, ©2024). The LPS structure consists of three main components: the O-antigen; the core oligosaccharide region, which is split into the L-Glycero-D-Manno-Heptose region (Hep) and the 3-deoxy-d-manno-oct-2-ulosonic acid (Kdo); and the lipid A region.

*Escherichia coli* (*E. coli*) is a gram-negative bacterium that lives harmlessly in healthy humans. However, pathogenic *E. coli* exists and harms immunocompromised humans. *E. coli* is implicated in many nosocomial infections, including septic shock, pneumonia, and meningitis. Worldwide, these infections affect over 100 million individuals per year. Therefore, studying the pathological mechanisms by which *E. coli* induces inflammatory responses during infection is relevant. This understanding will contribute to new knowledge and identify therapeutic targets or biomarkers for inflammatory disorders [1,2,3]. Bacterial lipopolysaccharides are the toxin components found on gram-negative bacterial cell walls. They are complex glycolipids comprising the O-antigen, the core oligosaccharide region, and diphosphoryl lipid A—the toxin (see Figure 1). The core oligosaccharide region can be further divided into the inner core (proximal to diphosphoryl lipid A) and outer core (proximal to the O-antigen), where the outer core is composed primarily of hexoses and the inner core is composed of the monosaccharide 2-keto-3-deoxyoctonoic acid (Kdo). The O-antigen is a chain of sugars that is highly variable and is responsible for the diversity of bacteria strains. The entire structure of bacterial lipopolysaccharide is called ‘smooth’, whereas ‘rough’ forms result from mutations that cause bacteria to lose their O-antigen [4,5,6,7,8].

Mammalian host immune response to bacterial infection comprises innate and adaptive immune responses. Innate immunity is the first line of defence against microbial infection or injury. It can be mediated by various cells, including macrophages, natural killer cells, neutrophils, dendritic cells, mast cells, eosinophils, basophils, and innate lymphoid cells. Their primary function is the elimination of microbial pathogens and foreign organisms via endocytosis/phagocytosis and enzymatic pathways. Some of these cells also present antigens from digested microbes to adaptive immune cells, linking the innate immune system to the adaptive immune system. Innate immune cells also secrete cytokines and chemokines, contributing to inflammation [9,10].

On a molecular level, pathogen recognition receptors drive innate immune cells’ recognition and inflammatory responses to microbial pathogens. They recognise pathogen-associated molecular patterns from microbes such as bacterial lipopolysaccharides and mediate inflammation via signalling pathways. Some commonly known pathogen recognition receptors include toll-like, nod-like, and RIG-I-like receptors. There are ten known toll-like receptors (TLRs) in humans, of which the last is a pseudogene. In other mammals, there are up to 13 toll-like receptors. TLR4 is the critical receptor for the recognition of bacterial lipopolysaccharides. It forms a complex with co-receptors—MD2 and CD14—known as the LPS receptor complex. Like other TLRs, TLR4 is synthesised in the endoplasmic reticulum before being transported to the plasma membrane, where it binds lipopolysaccharides [11,12,13].

The plasma membrane is a phospholipid bilayer that comprises various lipid species and proteins. Canonically, phospholipids found in the plasma membrane are phosphatidylcholine, sphingomyelin, cholesterol, gangliosides, phosphatidylethanolamine, and phosphatidylserine. The non-uniformity of the eukaryotic plasma membrane allows for protein trafficking, protein aggregation, the formation of microdomains, and signal transduction. Lipid rafts are detergent-resistant and highly dynamic plasma membrane microdomains. GPI-anchored proteins such as CD14 reside in lipid rafts. They play vital roles in biological processes such as endocytosis, protein trafficking, and signal transduction. Cholesterol is a pivotal component of lipid-raft microdomains as it keeps the lipid-raft assembly of sphingolipids and non-saturated phospholipids together. Therefore, cholesterol depletion in lipid rafts can lead to protein dissociation and dysfunction [14,15,16].

The phospholipid remodelling pathway maintains plasma membrane diversity by recycling lysophospholipids to phospholipids and vice versa. Substrates for this reaction come from the de novo pathway, and the key enzymes that participate in phospholipid remodelling are acyltransferases and phospholipases. LPCAT2 is one of these enzymes. It belongs to a group of LPCATs with similar structures and functions; however, *LPCAT2* is abundantly expressed in macrophages and participates in inflammatory processes [17,18,19,20]. Therefore, this study used gene expression analysis of crucial biomolecules for LPS recognition and macrophage inflammatory response to understand the mechanisms of LPCAT2 in inflammation. We compared groups of macrophages infected with smooth or rough serotype LPS to non-infected macrophages.

## 2. Materials and Methods

Chemical reagents, including nuclease-free water and DNase I amplification kit, were purchased from Sigma Aldrich, Gillingham, UK, and Fisher Scientific, Loughborough, UK. Pre-designed siRNA, Opti-MEM, Power SYBR Green, and the high-capacity RNA to cDNA kit were purchased from Life Technologies, Inchinnan, UK. PolyPlus Interferin and Lipofectamine 2000 reagents were purchased from PolyPlus-Sartorius, Epsom, UK. LAL reagent water, Dulbecco’s Modified Eagle Medium (DMEM), L-Glutamine, and Foetal Bovine Serum (FBS) were purchased from Lonza, Slough, UK. Smooth LPS—*E. coli* O111:B4—was purchased from Sigma-Aldrich. Semi-rough (Rc) LPS—*E. coli* J5 and Rough (Re) LPS—*E. coli* K12 D32m4—was purchased from List Biological Laboratories, Eastbourne, UK.

### 2.1. Cell Line and Culture

The RAW264.7-macrophage-like cell line was obtained from the European Collection of Cell Cultures (ECACC) through Public Health England, UK. RAW264.7 macrophages were maintained in DMEM supplemented with 10% (*v*/*v*) FBS and 1% (*v*/*v*) 0.2 M L-Glutamine, and incubated at 37 °C, 5% CO_2_. The cells were regularly monitored using inverted confocal microscopes, and cell viability checks were performed using trypan blue exclusion assays.

### 2.2. Gene Manipulation

RAW264.7 macrophages were cultured 24 h before gene manipulation. Then, a mixture containing PolyPlus Interferin transfection reagent, siRNA, and Opti-MEM as diluent was prepared and added slowly to a flask containing RAW264.7 cells. Finally, the cells were incubated at 37 °C, 5% CO_2_ with Opti-MEM for 24 h for efficient gene silencing.

Plasmid vector with or without murine LPCAT2 recombinant DNA was transfected into RAW264.7 cells using Lipofectamine 2000 transfection reagent, then incubated at 37 °C, 5% CO_2_ in Opti-MEM. Adding Geneticin to the flask containing RAW264.7 cells killed off those not taking up the plasmid vectors carrying *LPCAT2* and the neomycin-resistant gene, selecting only cells overexpressing *LPCAT2*.

### 2.3. Bacterial Infection

LPS was resuspended in LAL reagent water (<0.005 EU/mL endotoxin levels). Adding 100 ng/mL of either lipopolysaccharide serotype to a flask containing RAW264.7 cells stimulated the desired inflammatory response for 6 h, after which the culture medium was removed, and the cells were collected for analysis.

### 2.4. RNA Purification and Quantification

After bacterial infection, RAW264.7 cells were separated from the cell culture medium, rinsed with cold PBS (phosphate-buffered saline), and suspended in a buffer solution containing 4 M Guanidinium Thiocyanate, 0.75 M Sodium Citrate Dihydrate (pH 7.0), 10% (*w*/*v*) N-Laurosylsarcosine, and 0.72% (*v*/*v*) β-Mercaptoethanol for the RNA extraction procedure. The total RNA isolation procedure was adopted from [21] with minor modifications (chloroform was replaced with Bromochloropropane to form a liquid interphase). Total RNA was treated with DNase I to remove residual DNA according to the manufacturer’s protocol. Total RNA was quantified using NanoDrop2000 [ThermoFisher Scientific, Oxford, UK]; the purity was estimated using the A260/A280 ratio.

### 2.5. Primer Design

PCR primers were designed with Primer3 Plus Bioinformatics Software (version 3.3.0) and NCBI BLAST and purchased from Eurofins Genomics, Wolverhampton, UK. Selected primers had a low probability of primer dimer or secondary structure formation, GC% of 50–65%, and melting temperature between 55 and 60 °C.

### 2.6. Reverse Transcriptase-Real Time Quantitative Polymerase Chain Reaction

Following the manufacturer’s procedure for the high-capacity RNA to cDNA kit, we performed RNA to cDNA endpoint PCR. The reaction master mix for real-time quantitative PCR contained nuclease-free water, target primers (a mixture of forward and reverse primers), Power SYBR Green, and cDNA; the reaction was initiated at 95 °C for 10 min, then up to 40 repeated cycles of denaturing (15 s, 95 °C), annealing, and extension (60 s, 60 °C). *GAPDH* and *ATP5B* were used as endogenous controls.

### 2.7. Relative Quantitation and Statistical Analysis

We performed statistical analysis using R statistical programming software (version 4.3.2). Data represent the mean ± standard error of at least three independent experiments. We used paired *t*-tests with Dunnett’s multiple comparison tests to estimate statistical significance. All statistical tests were significant at a 95% confidence interval, *p* ≤ 0.05. We used the 2-ΔΔCt method [22] for the relative quantification of gene expression with *GAPDH* and *ATP5B* as endogenous reference genes.

## 3. Results

### 3.1. Structure of LPS

Bacterial lipopolysaccharides exist in various serotypes. The complete molecule that includes the ‘O-antigen’ polysaccharide repeat units is called smooth (S) LPS. In contrast, various truncated forms are known as rough (R) form LPS and have subtypes referred to as Ra, Rc, Rd, and Re, depending on the structure of the ‘core’ region of the molecule [5,6,7,8].

### 3.2. Analysis of LPCAT2 Transcription after Gene Silencing in Both Infected and Non-Infected RAW264.7 Macrophages

**Figure 2:**
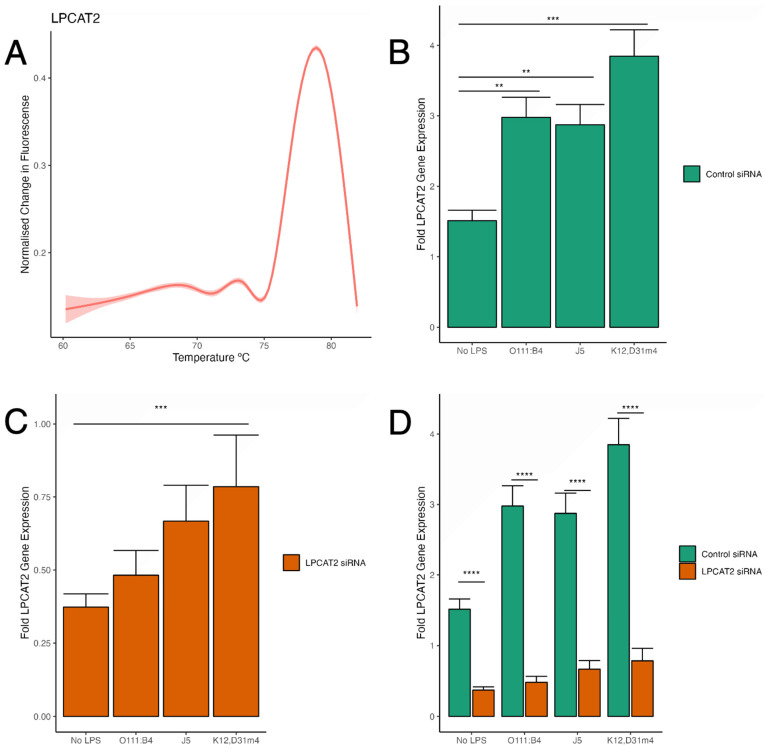
Efficiency of *LPCAT2* gene silencing in RAW264.7 macrophages with or without LPS infection. (**A**) The melt curve for the *LPCAT2* amplicon was obtained by reverse transcribing *LPCAT2* mRNA to cDNA and amplifying cDNA with real-time qPCR. Using R/ggplot2 (method = “gam”, formula = y~s (x, bs = “cs”)), the smooth curve was created from pooled data from all *LPCAT2* RT-qPCR experiments (≥3 independent experiments). (**B**) A control group of RAW264.7 macrophages were transiently transfected with negative siRNA and infected with either *E. coli* O111:B4, J5, or K12, D31m4 LPS serotypes; non-infected cells were used as negative controls. After incubation, the cells were lysed for RNA extraction and RT-qPCR. (**C**) A group of RAW264.7 macrophages was transiently transfected with *LPCAT2* siRNA and infected with either *E. coli* O111:B4, J5, or K12, D31m4 LPS serotypes; non-infected cells were used as negative controls. (**D**) *LPCAT2* gene expression in the control group is compared with that in the silenced *LPCAT2* group. Bars and error bars represent the mean ± standard error of the mean. **—*p* < 0.01, ***—*p* < 0.001, ****—*p* < 0.0001.

RT-qPCR was used to analyse *LPCAT2* gene expression to confirm the efficiency of the RNA interference gene silencing procedure in various conditions. Figure 2C,D show that the *LPCAT2* gene was significantly lower in macrophages transfected with *LPCAT2* siRNA with or without LPS infection. Furthermore, Figure 2B indicates that the *LPCAT2* gene was markedly higher in cells infected with LPS, but there was no significant difference between LPS serotypes. The melt curve in Figure 2A shows that the *LPCAT2* amplicon was specific, as there is only one peak above the noise level. Figure 2C shows a gradual increase in *LPCAT2* gene expression with each LPS serotype (smooth to rough), and macrophages infected with Re LPS had significantly higher *LPCAT2* gene expression than those without LPS. However, all values were below 1-fold, and there was no significant difference between LPS serotypes. Figure 2 shows ≥70% efficiency in silencing *LPCAT2* gene expression.

### 3.3. Differential Transcriptional Effect of Silencing LPCAT2 Gene on TLR4, CD14, and MD2 Gene Expression, Depending on LPS Serotype

**Figure 3:**
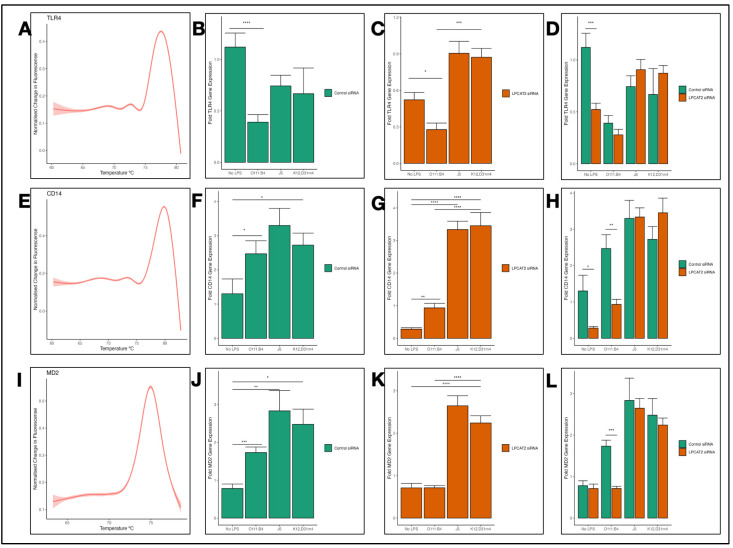
Transcriptional effects of silencing *LPCAT2* gene in RAW264.7 macrophages. (**A**,**E**,**I**) The melt curves for *TLR4*, *CD14,* and *MD2* amplicons were obtained by reverse transcribing mRNA to cDNA and amplifying cDNA with real-time qPCR. Using R/ggplot2 (method = “gam”, formula = y~s (x, bs = “cs”)), the smooth curves were created from pooled data from all *TLR4*, *CD14*, and *MD2* RT-qPCR experiments (≥3 independent experiments). (**B**,**F**,**J**) A control group of RAW264.7 macrophages were transiently transfected with negative siRNA and infected with either *E. coli* O111:B4, J5, or K12, D31m4 LPS serotypes; non-infected cells were used as negative controls. After incubation, the cells were lysed for RNA extraction and RT-qPCR. (**C**,**G**,**K**) A group of RAW264.7 macrophages was transiently transfected with *LPCAT2* siRNA and infected with either *E. coli* O111:B4, J5, or K12, D31m4 LPS serotypes; non-infected cells were used as negative controls. (**D**,**H**,**L**) *LPCAT2* gene expression in the control group is compared with that in the silenced *LPCAT2* group. Bars and error bars represent the mean ± standard error of the mean. *—*p* ≤ 0.05, **—*p* < 0.01, ***—*p* < 0.001, ****—*p* < 0.0001.

Using RT-qPCR, we analysed the transcriptional effects of silencing the *LPCAT2* gene on *TLR4*, *CD14*, and *MD2* gene expression in RAW264.7 macrophages. Figure 3A,E,I show that the amplicons were specific because they have a single fluorescence peak above the baseline. Figure 3B shows that infecting the macrophages with *E. coli* O111:B4 LPS caused downregulation of *TLR4* gene expression. However, macrophages infected with *E. coli* J5 or K12, D31m4 showed no significant change in *TLR4* gene expression. Furthermore, in negative controls (macrophages with no LPS infection), we see that silencing *LPCAT2* significantly decreased *TLR4* gene expression (Figure 3C). In macrophages infected with LPS, there was no significant difference in *TLR4* gene expression between the control and the silenced *LPCAT2* groups (Figure 3D). However, macrophages from the silenced *LPCAT2* group infected with *E. coli* O111:B4 had slightly lower *TLR4* gene expression than the control. *CD14* (Figure 3F) and *MD2* (Figure 3J) gene expression were significantly higher in LPS-infected macrophages. Like *TLR4*, *CD14* (Figure 3H) and *MD2* (Figure 3L) gene expression was not affected by silencing *LPCAT2*. However, macrophages from the silenced *LPCAT2* group showed significantly decreased gene expression of *CD14* (Figure 3G) and *MD2* (Figure 3K) compared to the control. This result indicates that silencing *LPCAT2* in non-infected macrophages decreased *TLR4* and *CD14* gene expression. Moreover, silencing *LPCAT2* in LPS-infected macrophages decreased *CD14* and *MD2* gene expression.

### 3.4. Overexpression of LPCAT2 in RAW264.7 Macrophages and Transcriptional Effect on TLR4 and CD14 Gene Expression

**Figure 4:**
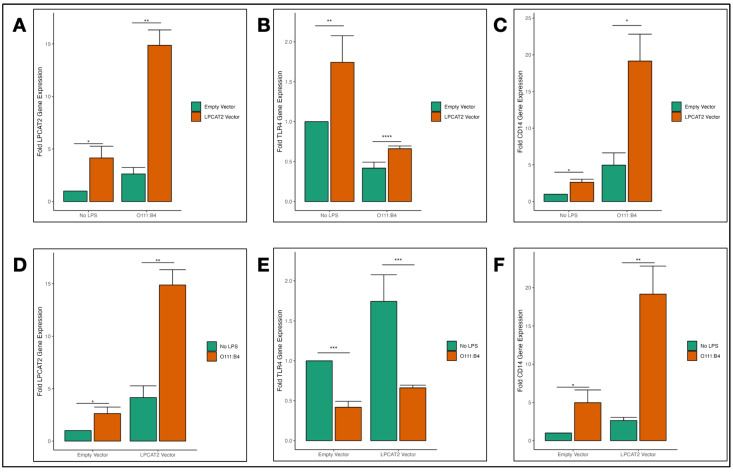
Transcriptional effects of overexpressing *LPCAT2* on *TLR4* and *CD14* gene expression in RAW264.7 macrophages. A control group of RAW264.7 macrophages were transiently transfected with either empty vectors (plasmid vectors with no target sequence) or *LPCAT2* vectors (plasmid vectors with *LPCAT2* sequence) and infected with either *E. coli* O111:B4, J5, or K12, D31m4 LPS serotypes; non-infected cells were used as negative controls. After incubation, the cells were lysed for RNA extraction and RT-qPCR. (**A**–**C**) Comparison of *LPCAT2*, *TLR4,* and *CD14* gene expression in non-infected and LPS-infected cells. (**D**–**F**) Comparison of *LPCAT2, TLR4*, and *CD14* gene expression in the control group and overexpressed *LPCAT2* group. Bars and error bars represent the mean ± standard error of the mean. *—*p* ≤ 0.05, **—*p* < 0.01, ***—*p* < 0.001, ****—*p* < 0.0001. Data were obtained from at least three independent experiments.

We overexpressed *LPCAT2* in RAW264.7 macrophages and used RT-qPCR to measure its effects on *TLR4* and *CD14* gene expression. Figure 4A,D show significantly higher *LPCAT2* gene expression in macrophages overexpressing *LPCAT2* with or without LPS infection, which confirms the efficiency of *LPCAT2* overexpression. Figure 3, above, shows that infecting RAW264.7 macrophages with *E. coli* O111:B4 downregulated *TLR4* gene expression, and Figure 4E shows a similar observation. However, on this occasion, macrophages overexpressing *LPCAT2* significantly increased *TLR4* gene expression with or without LPS (Figure 4B). On the other hand, *CD14* gene expression was markedly higher in macrophages overexpressing *LPCAT2* and infected with *E. coli* O111:B4 (Figure 4C,F). This result provides further evidence that *LPCAT2* regulates *CD14* and *TLR4* gene expression.

### 3.5. Differential Transcriptional Effect of Silencing LPCAT2 Gene on Tumor Necrosis Factor (TNFα), Interleukin 6 (IL6), Interferon Beta (IFNß), and Interferon Gamma-Inducible Protein 10 (IP10) Gene Expression, Depending on LPS Serotype

**Figure 5:**
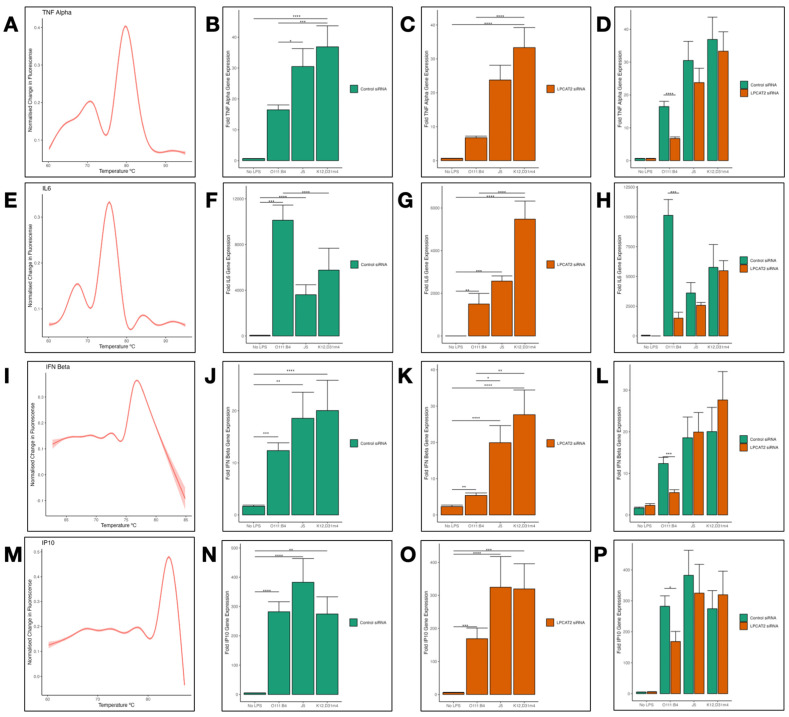
Transcriptional effects of silencing *LPCAT2* gene in RAW264.7 macrophages. (**A**,**E**,**I**,**M**). The melt curves for *TNF Alpha*, *IL6*, *IFN Beta*, and *IP10* amplicons were obtained by reverse transcribing mRNA to cDNA and amplifying cDNA with real-time qPCR. Using R/ggplot2 (method = “gam”, formula = y~s (x, bs = “cs”)), the smooth curves were created from pooled data from all *TNF Alpha*, *IL6*, *IFN Beta*, and *IP10* RT-qPCR experiments (≥3 independent experiments). (**B**,**F**,**J**,**N**) A control group of RAW264.7 macrophages were transiently transfected with negative siRNA and infected with either *E. coli* O111:B4, J5, or K12, D31m4 LPS serotypes; non-infected cells were used as negative controls. After incubation, the cells were lysed for RNA extraction and RT-qPCR. (**C**,**G**,**K**,**O**) A group of RAW264.7 macrophages was transiently transfected with *LPCAT2* siRNA and infected with either *E. coli* O111:B4, J5, or K12, D31m4 LPS serotypes; non-infected cells were used as negative controls. (**D**,**H**,**L**,**P**) *LPCAT2* gene expression in the control group is compared with that in the silenced *LPCAT2* group. Bars and error bars represent the mean ± standard error of the mean. *—*p* ≤ 0.05, **—*p* < 0.01, ***—*p* < 0.001, ****—*p* < 0.0001.

Using RT-qPCR, we analysed the transcriptional effects of silencing the *LPCAT2* gene on the gene expression of cytokines *TNFα*, *IL6*, *IFNß,* and *IP10* in RAW264.7 macrophages. The melt curves in Figure 5A,E,I,M show a single peak above the fluorescence baseline, indicating the specificity of PCR products. The effect of silencing *LPCAT2* on the cytokines in Figure 5 is akin to the influence on the gene expression of the LPS receptors. As expected, all serotypes of LPS induced high gene expression of the cytokines (Figure 5B,F,J,N). However, silencing *LPCAT2* only caused a significant in macrophages infected with *E. coli* O111:B4 (Figure 5D,H,L,P). Figure 5B shows a substantial difference in *TNFα* gene expression between macrophages infected with *E. coli* O111:B4 and J5/K12, D31m4.

## 4. Discussion

RAW264.7 macrophage cell lines are standard experimental models for studying inflammatory responses to various pathogen-associated molecular patterns. They were cloned from murine tumours using the Abelson leukaemia virus, and they express properties of macrophages such as phagocytosis, pinocytosis, and antibody-dependent pathogen killing [23]. They have also been used to study macrophage inflammatory responses to bacterial infection via various signalling pathways [24,25]. Although *E. coli* resides in healthy humans, there are pathogenic types that cause illness in immunocompromised humans. It is a frequent gram-negative bacterium that causes nosocomial infections, affecting more than 100 million patients annually worldwide. It also causes pneumonia, meningitis, and bacterial peritonitis [1,2,3].

The RT-qPCR experiments used SYBR Green as a fluorescent dye to quantify gene PCR amplicons in this study. SYBR Green is an intercalating dye that fluoresces when it binds to the minor groove of a double-stranded DNA [26]. Melt curves often validate the purity or specificity of amplicons from PCR assays using intercalating dyes, as specificity is usually a concern for these assays. It is generally assumed that more than one peak in the melt curve indicates the presence of more than one amplicon, as different amplicons will have different melting temperatures. However, that is only sometimes valid [27,28].

The broad premise of this study was to show that LPCAT2 is a pro-inflammatory molecule that regulates macrophage inflammatory responses in an LPS-serotype-dependent manner. Figure 2 and Figure 5 show that *LPCAT2* gene expression was increased in macrophages infected with LPS. This observation is in line with reports from several other publications. Besides *LPCAT2* gene expression, its protein expression and enzymatic activity are enhanced during infection [17,29]. Various evidence-based theories exist for the molecular mechanisms by which *LPCAT2* influences macrophage inflammatory responses. It could be via its metabolites [19,30], by association with the LPS receptor—TLR4—during the inflammatory response to bacterial infection [11], or by influencing post-translational modifications during the inflammatory response to bacterial infection [18]. Phospholipid remodelling is crucial for forming lipid rafts (phospholipid membrane ‘platforms’ for signalling), and LPCAT2 participates in phospholipid remodelling; this is also one of the proposed mechanisms.

Moreover, LPCAT2 has been implicated as a biomarker for inflammatory disorders such as cedar pollen allergenic rhinitis [31], pneumonia [32], and pulmonary tuberculosis [33]. All these publications support evidence that LPCAT2 is pro-inflammatory; however, data on the effect of LPCAT2 on various pro-inflammatory molecules are unavailable.

TLR4, CD14, and MD2 are crucial for macrophage inflammatory responses to LPS [34]. CD14 first recognises and binds to LPS, then presents it to the TLR4–MD2 complex. It is a co-receptor for other TLRs; therefore, it can mediate inflammation without TLR4 [35]. On the other hand, MD2 associates with the extracellular domain of TLR4 and enhances its response to LPS recognition. It also plays a role in TLR4 subcellular localisation [36,37]. TLR4 has two canonical signalling pathways: the MyD88-dependent and MyD88-independent pathways. Scientific evidence shows CD14 is vital for TLR4 signalling via the MyD88-independent pathway [38]. As the results described in this paper confirm, *CD14* gene expression is increased by LPS in alveolar macrophages after several hours of infection [39], *TLR4* gene expression is decreased by LPS infection of mouse macrophages [40], and *MD2* gene expression is higher after LPS infection of alveolar macrophages [41]. Furthermore, our data show, in Figure 3 and Figure 4, that *LPCAT2* regulated these molecules’ gene expression, albeit only in non-infected and *E. coli* O111:B4-infected macrophages. This is a novel finding, as no other publication has shown the effect of *LPCAT2* on *TLR4*, *CD14,* and *MD2* gene expression. Previous studies mentioned that LPCAT2 did not influence CD14 or TLR4 expression; however, no data was shown, nor was the type of molecular expression clarified. Lysophosphatidic acid (LPA) is a product of LPCAT2 metabolite—lysophosphatidylcholine [42]. LPA1, a receptor that recognises and binds LPA and LPS, was shown to regulate CD14 expression in epithelial cells [43]. This somewhat supports the results in Figure 3 and Figure 4. Moreover, several publications have demonstrated that *CD14, TLR4,* and *MD2* gene expression decreases when the inflammatory response to LPS infection is inhibited [44,45].

Downstream of TLR4 signalling, transcription factors such as Interferon Regulatory Factor (IRF3), Nuclear Factor Kappa Beta (NFκB), and Mitogen-Activated Protein Kinase (MAPK) proteins induce the transcription of cytokines, which leads to cytokine release and, thus, feeds back into the inflammatory response [46]. Interferon beta is known to be favourably induced by the TLR4-MyD88-independent pathway by IRF3 [47]. On the other hand, *IL6*, *TNFα*, and *IP10* are induced by NFκΒ [48]. In Figure 5, our results show that all serotypes of LPS increased cytokine gene expression, and LPCAT2 regulated cytokine expression. Several other studies have shown similar evidence [11,14,22,25]. However, we show here, for the first time, that LPCAT2 regulates *IFNß*.

Researchers have shown evidence that various LPS serotypes differentially regulate *TNFα* gene expression. Rough Salmonella and Brucella serotypes induce higher *TNFα* and *IL6* expression in monocytes and macrophages [49,50]. This supports the data shown in Figure 5B. Our results, however, show higher *IL6* expression with smooth LPS (Figure 5F). Conversely, Figure 5N shows no significant difference in *IP10* gene expression between macrophages infected with smooth or rough LPS. A study that compared various serotypes of *E. coli* and Brucella LPS showed similar results [51].

Many species of bacteria have either smooth or rough LPS serotypes, and these variances in bacterial structures cause differences in their pathological mechanisms. Rough forms of LPS can activate the TLR4-MyD88-dependent signalling pathway without *CD14* [52,53,54], and *CD14* is crucial for endocytosis, which depends on lipid-raft formation [15,16,55]. Furthermore, there is evidence that smooth forms of LPS rely on lipid-raft formation for internalization, but not rough forms. In J774.A1 cells, disrupting lipid-raft formation prevented the internalisation of smooth Brucella LPS but had limited effect on rough Brucella LPS [56]. Cholesterol is an essential component of lipid rafts. However, high-density lipoprotein interacts with high-density lipoprotein receptors, leading to lipid-rafts’ cholesterol depletion. Cholesterol depletion in lipid rafts leads to protein dissociation and dysfunction [15,57]. Researchers have shown that high-density lipoprotein caused decreased binding and uptake of smooth-type LPS in CHO cells [58]. In the same vein, the results of this study show that *LPCAT2* did not influence inflammatory response to rough LPS. Although CD14 is required for LPS endocytosis, rough LPS is still endocytosed, suggesting it may use a CD14-independent endocytosis pathway. The phagocytic pathways of rough and smooth LPS are different, where phagosomes containing rough LPS are fusiogenic, phagosomes containing smooth LPS are non-fusiogenic. Also, rough LPS activates the inflammasome, Ca2+/calcineurin, and NFAT pathways in dendritic cells, leading to higher cytokine production than smooth LPS [49,52]. As LPCAT2 affects CD14-dependent endocytosis, this published evidence aligns with our finding that rough LPS induced higher cytokine production but was not affected by LPCAT2 as it is independent of CD14. Overall, this supports the theory that LPCAT2 influences macrophage inflammatory responses by regulating lipid-raft formation and supports the previous study that showed evidence of LPCAT2 inhibition preventing TLR4 translocation to lipid rafts [17].

Lipidomic, proteomic, and genomic analysis would be beneficial for better understanding the influence of LPCAT2 on macrophage inflammatory response to bacterial infection. Due to limited research funds, we selected key molecular targets for this study. Additionally, it will be advantageous to study the influence of LPCAT2 on macrophage inflammatory responses mediated by other pathogen recognition receptors that either depend on CD14 or require lipid-raft domains for signalling. As this study was carried out in vitro using murine secondary cell lines, replicating it in human primary cell lines or ex vivo studies would be favourable because they would imply applicability in vivo. A better understanding of the influence of LPCAT2 on phagocytic pathways and cell morphology would also be beneficial. An in-depth understanding of LPCAT2 molecular mechanisms in macrophage inflammatory response will make it a potential therapeutic target or biomarker for inflammatory disorder.

## 5. Conclusions

In conclusion, this study has shown novel evidence that LPCAT2 does not influence macrophage inflammatory responses to rough forms of *E. coli*. We have also published new evidence that LPCAT2 regulates *CD14*, *TLR4*, *MD2*, and *IFNß* gene expression. The ability of *LPCAT2* to regulate *CD14* gene expression implies that it influences CD14-dependent endocytosis, which is crucial for TLR4 signalling. As lipid-raft domains are vital to endocytosis, it also indicates that LPCAT2 influences the assembly of lipid-raft domains. Several publications suggest that rough forms of LPS do not require lipid-raft domains for mediating inflammatory responses. This aligns with our finding that LPCAT2 only influences macrophage inflammatory response to smooth-form LPS because LPCAT2 participates in the phospholipid remodelling pathway, which regulates the assembly or depletion of lipid-raft domains. Our research group has previously shown that silencing *LPCAT2* prevents TLR4 translocation to the lipid-raft domain, an observation supported by this study and another study that showed cholesterol depletion did not influence rough LPS uptake but influenced smooth LPS uptake.

Simply put, this study provides evidence that LPCAT2 does not influence macrophage inflammatory responses to rough forms of LPS and supports previous evidence by our research group that LPCAT2 regulates macrophage inflammatory responses by influencing TLR4 translocation to lipid-raft domains. These results will pave the way for further studies to better understand the role of LPCAT2 in inflammatory responses. It also contributes insights to biochemistry, immunology, and molecular biology.

## Data Availability

Data are available upon request.
